# A two cases clinical report of mandragora poisoning in primary care in Crete, Greece: two case report

**DOI:** 10.1186/1757-1626-2-9331

**Published:** 2009-12-16

**Authors:** Ioanna G Tsiligianni, Theodoros K Vasilopoulos, Polyvios K Papadokostakis, Georgia K Arseni, Astrinaki Eleni, Christos D Lionis

**Affiliations:** 1 Agia Barbara Primary Health Care Centre, Agia Barbara, Heraklion, Crete, PO 70003, Greece; 2 Archalohori Primary Health Care Centre, Archalohori, Heraklion, Crete, PO 70300, Greece; 3 Intensive care unit of Venizeleion hospital, Venizeleion hospital, Heraklion, Crete, PO 71001, Greece; 4 Clinic of Social and Family Medicine, School of Medicine, University of Crete, Heraklion, Crete, PO 71003, Greece

## Abstract

**Introduction:**

People in Greece, especially those living in rural areas, frequently consume various plants and herbs as a vegetable meal or as a herbal remedy, which can lead to a number of adverse reactions. These two case reports resulted in a prolonged hospitalisation due to severe and persistent supraventricular tachycardia caused by a vegetable meal.

**Cases presentation:**

These case reports describe two cases of accidentally use of Mandragora Officinarum identified within the same Greek family, which resulted in hospitalisation. A 47-year-old Greek Caucasian woman and a 48-year-old Greek Caucasian male presented to the local primary care centre with nausea, dizziness, blurred vision, headache and dryness of mouth. Due to serious supraventricular tachycardia, the two patients were hospitalised in the intensive care unit of a nearby hospital for a week.

**Conclusion:**

These case reports highlight the importance of ensuring that primary care physicians are aware of the possible effects of mandragora use, for cases when they are involved in the treatment of patients presenting with similar symptoms as those discussed below.

## Introduction

People, especially those living in rural areas, frequently collect plants and herbs grown near their home for consumption as a vegetable meal, or as a herbal remedy [1]. Mandragora is a solanaceous plant, native in the Mediterranean and southern and central Europe. The plant can be found in Greece, particularly on Crete and Amorgos island. The best-known species, Mandragora Officinarum, belongs to the nightshade family, and it has a short stem bearing ovate pale blue or violet flowers. The plant has a thick, fleshy, often-forked root, and for this reason it is thought to resemble a human figure. Large leaves are also present, together with round berries known as 'devils apples'. All parts of the plant are strongly sedative.

The high concentration of solanum alkaloids and tropane alkaloids, present in mandragora, gives rise to anticholinergic properties when it is consumed as a vegetable. These anticholinergic properties can cause severe symptoms such as nausea, mydriasis, blurred vision and supraventricular tachycardia. Scopolamine can cause serious central nervous findings, more often presents with an excited or agitated delirium and less often with recent amnesia and suppression of the central nervous system. Up to the present time, more than 80 substances are isolated in different species of the genus Mandragora [2].

## Cases presentation

A 47-year-old Caucasian Greek woman and a 48-year-old Caucasian Greek male from the same family were brought to the emergency services of Agia Barbara primary care health centre, on the island of Crete, Greece. Both patients exhibited a number of symptoms, which included nausea, dizziness, blurred vision, dryness of mouth, headache, instability in walking and vomiting. The reported symptoms raised a number of diagnostic questions, and the patients were asked what they had eaten. The male patient reported consumption of a plant as a vegetable meal. We asked him to bring the plant to us, which we identified via figures found after an Internet search. The two patients consumed accidentally mandragora instead of the commonly used eatable borago officinalis.

When vital signs were measured, both patients were normotensive with normal temperature and oxygen saturation, but with raised pulse (190 beats per minute for the man and 170 for the woman) and respiratory rate (25 breaths per minute for the man and 20 for the woman). Flushed skin and reactive mydriasis were recorded. The ECG revealed in both patients supraventricular tachycardia (180 heart rate for the man and 173 heart rate for the woman).

Treatment was aimed first at removing plant material from the gastrointestinal tract, with a gastric lavage and secondly towards the administration of cholinergic agents. Both patients took 1 mg of physostigmine IV per day as treatment. The patients didn't take any benzodiazepines because the neurologic symptoms were not persistent contrary to the cardiovascular symptoms. In details the walking instability, the blurred vision, the headache and the mydriasis remained only for the first two days. Because of the serious symptomatology at the beginning and the severe and the persistent supraventricular tachycardia, the two patients were hospitalised within 2 hour after the consumption of the vegetable meal in the intensive care unit of the nearest secondary care hospital at Heraklion city. Laboratory tests were performed at the Hospital's Emergency Department revealed a white blood cell count, urine and blood screening within normal limits. Only the liver transaminase tests such as aspartate transaminase (AST) and alanine transaminase (ALT) were raised (AST 75 U/L and ALT 65 U/L for the man, and AST 63 U/L and ALT 55 U/L for the woman). The liver transaminase tests were returned within normal limits in approximately 10 days. As the hospitals physicians reported, the patients were discharged from the hospital approximately one week after initial admission. The neurologic symptoms previous mentioned were present in both patients only for the first two days. The reason that they stayed for so long in the hospital was mainly the persistent supraventricular tachycardia. The patients responsed well to the use of physostigmine and the supraventricular tachycardia was set up after 5 days. The final diagnosis was toxication caused by mandragora use.

## Discussion

Á systematic search performed in electronic databases, (from 1970 to 2008) including medline, embase, pubmed and the Cohrane Library identified at least 24 clinical cases due to mandragora poisoning.

Since antiquity, mandragora has been used as a sedative and aphrodisiac, and it was believed by some to have magical powers. It was utilised as an analgesic, an emetic and also as an anesthetic for surgical purposes [3]. It has significant emetic and cathartic effects, inducing contractions of the stomach, and as such has been associated with the delusion that could help in child-bearing. The Greek herbalist Dioscorides provides a detailed description of mandragora and its use [4,5]. He further reports the hypnotic effects of a preparation of mandragora in wine, administered to patients undergoing anesthesia [3,4].

The differential diagnosis included also other plants that could cause similar atropine poisoning effects such as Jimson weed or Podophylum peltatum and the use of drugs with anticholinergic side effects such as tricyclic antidepressants. The mandragora is consumed by a number of people residing in rural areas either accidentally (as a vegetable meal), or as a medicinal herb, with the consumer being unaware of the adverse reactions [1,2,6]. Jimenez-Mejias and colleagues identified 15 cases of mandragora poisoning in Spain [7]. All patients reported with blurred vision and dryness of mouth, whilst a number of individual symptoms were identified including micturation difficulties, dizziness, headache, vomiting, difficultly swallowing, and abdominal pain [7]. Blushing, areactive mydriasis and dysrythmia were found in all 15 cases, dry skin, mucosae, hyperactivity/hallucination was identified in 14, and 9 of the patients presented with agitation/delirium. In this case series all patients were treated with either prostigmine or physostigmine with a better reversal of symptoms in those taking physostigmine [7]. Likewise, Piccillo et al, identified 6 clinical cases of mandragora poisoning in patients reporting with similar symptoms in Sicily, leading them to recommend a number of possible treatments including vital function support [8]. A further paper by Piccillo et al, reports on a case of anticholinergic syndrome due to 'Devil's herb', identified in a 72 year old female who had mistakingly consumed mandragora believing it to be the edible *borago officinalis *[9]. An earlier report by De Salvo and colleagues following identification of mandragora poisoning in a single patient draws attention to involvement of considerable liver and kidney dysfunction [10].

It is known to the authors that there are already similar case reports of mandragora poisoning in the literature but to our knowledge this is the first paper which reports on two simultaneous cases of hospitalisation in an intensive care unit, due to severe and prolonged supraventricular tachycardia caused by the consumption of Mandragora Officinarum in Greece. Although the main weakness of this report is that no confirmatory test was performed to either confirm the plant or to exclude other coingestants, it highlights the importance of ensuring that primary care physicians mainly serving rural areas are aware of the toxic effects arising from the consumption of mandragora and other poisonous plants/medicinal herbs.

## Conclusion

These unusual presentations of persistent supraventricular tachycardia as a complication of the toxicity of a native plant consumed accidentally as a vegetable meal highlights the need for high index of suspicious mainly for primary care physicians that serve rural areas. A further issue of concern relates to the fact that patients are often unaware of the toxicity of certain plants, or may consume them as a result of mistaking them for alternative edible herbs. Questioning patients, with regard to recent consumption of such plants may assist physicians in their diagnosis, thus avoiding delays lead to serious complications.

## Consent

Written informed consents were obtained from the patients for publication of these two cases clinical report. A copy of the written consents are available for review by the journal's Editor-in-Chief.

## Competing interests

The authors declare that they have no competing interests.

## Authors' contributions

IT, TV, GA, PP, AE analysed and interpreted the patients data. IT, TV, GA, PP, AE searched the literature for similar cases. IT and CL were major contributors in writing the manuscript. All authors read and approved the final manuscript.
